# Expression and high levels of insertional polymorphism of an endogenous gammaretrovirus lineage in dogs

**DOI:** 10.1371/journal.pgen.1011083

**Published:** 2023-12-06

**Authors:** Abigail S. Jarosz, Amanda L. Pendleton, Michael J. Lashbrook, Erica Cech, Madison Altieri, Austin Kunch, Jaime F. Modiano, Julia V. Halo

**Affiliations:** 1 Bowling Green State University, Department of Biological Sciences, Bowling Green, Ohio, United States of America; 2 Purdue University, Department of Biochemistry, West Lafayette, Indiana, United States of America; 3 Purdue University, Purdue Center for Plant Biology, West Lafayette, Indiana, United States of America; 4 Animal Cancer Care and Research Program, University of Minnesota, St. Paul, Minnesota, United States of America; 5 Department of Veterinary Clinical Sciences, College of Veterinary Medicine, University of Minnesota, St. Paul, Minnesota, United States of America; 6 Masonic Cancer Center, University of Minnesota, Minneapolis, Minnesota, United States of America; 7 Center for Immunology, University of Minnesota, Minneapolis, Minnesota, United States of America; 8 Stem Cell Institute, University of Minnesota, Minneapolis, Minnesota, United States of America; 9 Institute for Engineering in Medicine, University of Minnesota, Minneapolis, Minnesota, United States of America; 10 Department of Laboratory Medicine and Pathology, Medical School, University of Minnesota, Minneapolis, Minnesota, United States of America; Texas A&M University College Station, UNITED STATES

## Abstract

Despite the absence of a confirmed exogenously replicating retrovirus in *Canis lupus familiaris* (*C*. *familiaris*), past retroviral infections are evident in the genomes of living animals via the presence of endogenous retroviruses (ERVs). Although gammaretrovirus-like transcripts and enzyme activities were previously reported to be present in canine leukemias and lymphomas, those findings were not further explored. Initial analysis of the *C*. *familiaris* reference genome revealed a minor subset of one ERV lineage, classified as CfERV-Fc1(a), or Fc1(a) here, with features characteristic of recent integration, including the presence of ORFs and identical or nearly identical LTRs. Our previous analysis of whole genome sequence data belonging to extant Canidae revealed a burst of past infections in *Canis* ancestors resulting in numerous young, polymorphic, and highly intact loci now segregating in dogs. Here, we demonstrate the expression of full-length Fc1(a) proviruses in tissues collected from healthy animals and from animals with cancer. We observed significantly higher expression in samples of dogs with various cancer diagnoses when compared to samples from healthy dogs. Genotyping of insertionally polymorphic Fc1(a) loci identified candidate expressed proviruses and delineated distributions over sample groups. Collectively, the data show that Fc1(a) proviruses retain biological activity in the domestic dog and provides a means to examine potential genetic links with disease states in this species.

## Author summary

The domestic dog is an important biomedical model of human genetics and disease. Until now, the dog has been largely excluded from research focusing on retrovirus biology and retrovirus-host coevolution in the context of the canine as a host. Our work reveals that numerous recently integrated endogenous retroviruses are variably present in the genomes of domestic dogs including ones that are transcriptionally active or are present within or near genes, and that transcriptional activity is significantly elevated in tumors of various canine cancers. Thus, our work characterizes a previously unexplored resource of genomic impact to the domestic dog as a model of health and disease. The results from this study begin span a significant knowledge gap in our understanding of retrovirus-host dynamics in the dog as a biomedical model of genetics and improve our understanding of the biological activity and prevalence of a zoonotic-relevant and highly intact endogenous retrovirus lineage in the domestic dog.

## Introduction

The replication cycle of retroviruses is unique among mammalian viruses in the obligatory step of integrating a chromosomal copy of the infecting viral genome as a provirus. Consequently, infection of germline DNA may lead to a provirus that is transmitted to the host’s offspring, referred to as an endogenous retrovirus (ERV) [1]. Following integration, a canonical full-length ERV contains the viral open reading frames (ORFs; *gag*, *pro*/*pol*, and *env*) flanked by regulatory segments referred to as long terminal repeats (LTRs; 5’LTR, 3’LTR) that are identical upon integration and contain regulatory elements that direct proviral transcription [2–4]. The vast majority of ERVs have lost infectious capacity due to accumulated changes, indels, or truncation (the latter referred to as ‘near’ full-length), or from recombination between the proviral LTRs resulting in a solo-LTR. ERVs have contributed to abundant proportions of many species’ genomes [1,5]. For example, over 8% of the human genome is recognizably of retroviral origin. The genomes of several species, including human, are known to harbor ERV lineages with evidence of recent or ongoing germline invasion [5]. Members of these lineages appear more intact and are include ‘young’ insertions, as evidenced by insertional polymorphism (*i*.*e*., the presence of unfixed loci of ‘insertion’ as well as ‘empty’ alleles in the sampled population), high sequence identity between integrants, the presence of one or more open reading frames (ORFs), or encoded function(s) [6], raising the possibility of retained viral gene functions or LTR derived regulatory properties.

Previous analyses of tumors and affected tissues of immunosuppressed representatives of the domestic dog (*Canis lupus familiaris*) led to several reports of observations of retrovirus-like associated products. For example, retrovirus-derived RNAs and reverse transcriptase enzyme activities were isolated from tumors, and supernatant filtrates that contained particles of C-type morphology consistent with a γ-like retrovirus were observed in dogs with cancers including leukemia [7–11], lymphoma [12,13], as well as other malignancies or severe immunosuppression [14–16]. An explanation stemming from these works was the existence of a pathogenic canine retrovirus capable of contributing to cancers or immune suppression as was known to occur in other species [14]. However, possible contamination of tissues by infectious retroviruses from other species was not excluded following those reports, and there has paradoxically never been a confirmed infectious retrovirus in dogs. An alternative explanation for the presence γ-like retroviral products could be offered by the deregulation of ERVs resulting in their transcription or subsequent translation of encoded products therein. In this regard, several mammalian species harbor ERV lineages that include members with retained biological activities or even the ability to produce infectious virions, including but not limited to humans [1], mice [17], and cats [18].

**Fig 1:**
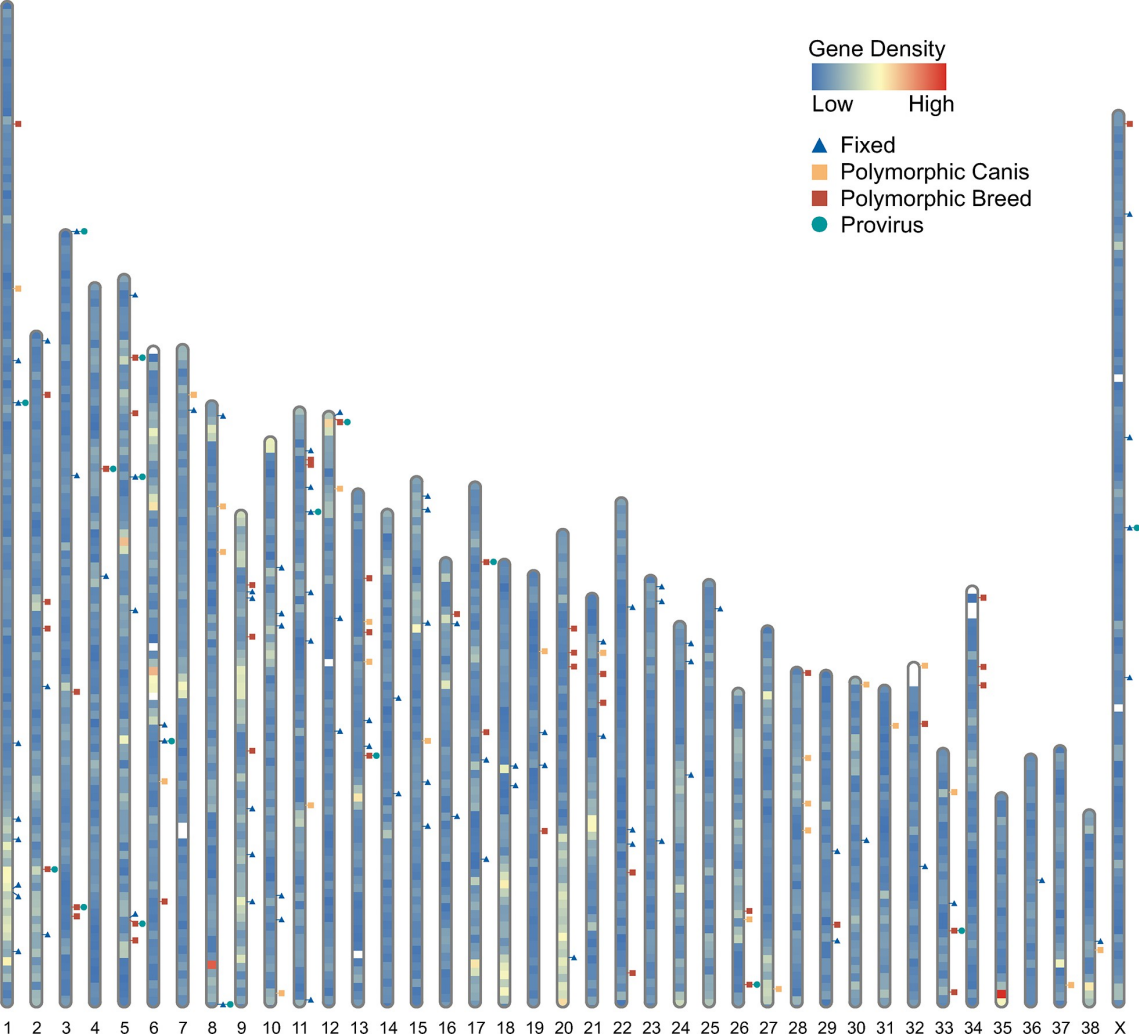
Genomic distribution of the Fc1(a) ERV lineage. Chromosome representation of Fc1(a) insertion loci and gene density mapped to the CanFam3.1 boxer genome build. Coordinates corresponding to Fc1(a) insertions and status of insertional polymorphism are as previously identified by Halo and colleagues [21]. Gene density, karyotype, and marker files from NCBI [19] were used to generate gene karyotype and density using a personal python script. The output files were implemented to RIdeogram [37] to visualize gene density over individual chromosomes, excluding unplaced contigs (chrUn) from analysis. Dark blue triangles show placement of insertions that are fixed among *Canis spp*.; dark red squares denote insertions previously deemed as insertionally polymorphic among modern breeds; orange squares mark additional insertions that are insertionally polymorphic in wild canids; teal circles show loci for which a provirus allele has been confirmed. Gene density is represented by a heatmap with gene poor regions in blue and gene dense regions in red.

Based on initial analysis of the *C*. *familiaris* reference genome assembly from a female boxer breed dog, ‘Tasha’, (first released in 2005) [19], ~3.6% was ERV-derived [19], later estimated to include ~0.15% from full-length or near full-length proviruses [20]. Analysis of that genome build revealed the majority of ERVs were inactivated and estimated to have colonized dog ancestors in times ranging from roughly 12.5 to 25 million years ago (mya). However, two γ-like full-length ERV-Fc-related proviruses (so-named for inferred use of a tRNA^phe^ to prime reverse transcription) were identified with putative ORFs and low LTR-LTR divergence and were therefore presumed to be considerably younger [20]. The significance of these observations was previously uncovered in a comparative analyses of available whole genome data of domestic and wild canids against the CanFam3.1 reference build, leading to discoveries of numerous young loci and recent bursts of germline infections from members of an ERV-Fc related lineage, ‘CfERV-Fc1(a)’ (Fig 1) [21]. For brevity we refer to this lineage as ‘Fc1(a)’ throughout this text. The Fc1(a) ERV lineage first entered the canid germline prior to the split from the red fox (*Vulpes vulpes*) ~12 mya [22] and continued to infect dog ancestors until within the last ~400ky [21]. The invading exogenous form was a recombinant virus derived from the ERV-Fc (5’ LTR, *gag*, *pro*/*pol*, 3’LTR) and distantly related ERV-W (*env*; human syncytin-like) γ-like groups, likely having originated from co-packaging of RNAs from unrelated source proviruses in the infecting virion [22]. The Fc1(a) ERV-W *env* is predicted as belonging to the RD114-and-D-type-retrovirus (RDR) interference group [23]; its acquisition likely contributed to altered pathogenic properties of the Fc1(a) progenitor, thus facilitating transmission to canid ancestors [22].

Our previous characterization of Fc1(a) loci in the genomes of modern breeds, as well as orthologous comparisons of representative genomes of all living *Canis spp*., revealed that past spread of this lineage has contributed to numerous polymorphic loci in contemporary animals (Fig 1). For example, nearly half of 145 genotyped loci, or 46.2%, were found to be insertionally polymorphic across *Canis spp*., including 40 loci that were deemed as variably present in modern breeds [21]. These insertions tended to have short branch lengths and low group divergence (S1 Fig). Nineteen full-length or near full-length proviruses have been currently annotated and include full-length members predicted to have at least one ORF, as well as multiple insertions of identical sequences between 5’ and 3’ LTRs [21]. Proviruses with the shortest branch lengths tended to possess an uninterrupted *env* ORF (bold envelope symbols in S1 Fig), implying its function in Fc1(a) spread. The LTRs of the youngest full-length insertions also tended to pair on sister branches, and subsets shared identical LTR sequences with solo-LTRs. These collective lines of evidence point to recent infectious capacity and raise the possibility that replication-competent Fc1(a) viruses recently (and may presently) exist within living animals, or have the capability to facilitate ‘breakout’ of an infectious recombinant [24].

ERVs are known to impact both genome structure and physiological functions of the host. In this regard, the most recent integrants, or those not yet fixed within a host species, should have the most potential for biological impact having been recently introduced to the genome and not yet subject to long-term evolutionary pressures. Thus, the highly variable presence and recent addition to the domestic dog genome also lends candidacy for Fc1(a) loci possessing such properties for interference of normal gene regulation or aberrant transcriptional effects due to their integration within ‘new’ genomic contexts, noting many are situated within genes or regions proximal to predicted dog gene models [21]. For example, of the annotated repertoire of Fc1(a) insertions, roughly 44% (69 loci) are within 25kb of an Ensembl dog gene model; roughly 18% are located within introns of genes (27 loci) and one is present in the 3’ untranslated region of a gene exon [21].

Given the relatively recent formation, the presence of viral ORFs, and ‘intactness’ of members of this lineage, we therefore asked if Fc1(a) retained the potential for biological activity. In this regard, the deregulation of the Fc1(a) lineage would lend itself a likely candidate for the previously reported γ-like retroviral activities in canine leukemias, lymphomas, or other malignancies. In this study, we provide evidence for expression of Fc1(a) derived sequences in total RNAs from canine cell lines as well as total RNAs isolated from distinct tissues from healthy dogs. By focusing on *env*-derived transcripts, we show that expression from Fc1(a) proviruses is also detected in tumor tissues from dogs diagnosed with chronic and acute leukemias or lymphomas, as well as melanomas, osteosarcomas, and others. The detection of sequences corresponding to Fc1(a) *env* was significantly elevated in tumor tissues as compared to blood samples from healthy dogs. Genotyping of Fc1(a) insertions in dogs of modern breeds, including near or full-length proviruses and solo-LTRs, indicates candidate source proviruses of this expression, and delineates the prevalence of insertionally polymorphic Fc1(a) loci across samples as well as their presence in the context of genic locale. As ERV presence and biological activities in other species are known to impact genomic variation and disease this work highlights the possibility of similar consequences in dogs.

## Results

### The *env* gene is most present among annotated Fc1(a) proviruses

Of the proviruses, 11 are present within the CanFam3.1 reference and 8 additional insertions were obtained in non-reference discoveries briefly described above [21] (S1 Table). A nucleotide alignment of the 19 proviral integrants characterized in *Canis spp*. yielded an inferred consensus, Fc1(a)_CON_ [21,25] (S2 Fig). Briefly, it contains complete open readings frames (ORFs) for *gag*, *pro*/*pol* and *env*, putatively intact structural regions and motifs that would be necessary for replication competency, a tRNA^Phe^ primer binding site, and identical 5’ and 3’ LTR segments. Thus, in principle, few changes would be necessary to generate a putatively replication competent virus.

We assessed the 19 currently known Fc1(a) proviruses for the presence of reading frames as well as motifs therein that would be predicted as necessary for viral gene product function (summarized in S1 Table). The *env* reading frame was the most frequently present of the viral genes among the Fc1(a) full-length integrants; seven of the 19 possessed clear *env* ORFs, whereas six displayed a putative *pro*/*pol* ORF and none possessed an intact *gag*. The seven *env* genes ranged from 98.9% (insertion at chr6:47,934,941) to 99.8% (chr12:869,873) nucleotide identity to the Fc1(a)_CON_ *env*; the translated Env products are highly similar in sequence to the Fc1(a)_CON_, sharing from 98.4% (9 changes, insertion at chrX:50,661,637) to identical (chr12:869,873) amino acid content. Aside from the chrX provirus, all but one possessed apparent unaltered sequence motifs required for function (chr5:10,128,780; RRKR➔WRKR in its furin cleavage site), including identical RDR-like motifs predicted to be involved in receptor interactions, and at most 3 changes in the translated amino acid sequence (chr13:32,380,539); the chrX Env has incurred changes in predicted ISD and TM motifs [21]. Due to its conservation and apparent intactness in the majority of the most recently integrated proviruses, we therefore examined expression of Fc1(a) *env* in canine cell lines and tissues.

### Fc1(a) transcripts are detected in canine cell line RNAs

To first establish our approach for detection and measuring expression, we investigated the presence of *env*-containing transcripts over total RNAs isolated from canine cell lines of A72 fibrosarcoma, DH82 histiocytic sarcoma, D17 osteosarcoma, and MDCK derived from healthy kidney. Utilizing conserved regions within the Fc1(a)_CON_ *env* ORF, primers were designed to amplify a 300 bp region within the SU domain (see Methods, S2 Fig). The selected region is absent from 10 of the 19 annotated proviruses, which instead either possess a common deletion of 1073 bp (*env*_Δ1073bp_; eight of the 10) or another *env* deletion disrupting *env* (two of the 10; also refer to S1 Table). Thus, our analysis was limited to an expressed *env* region present in annotated and not-yet annotated Fc1(a) proviruses.

**Fig 2:**
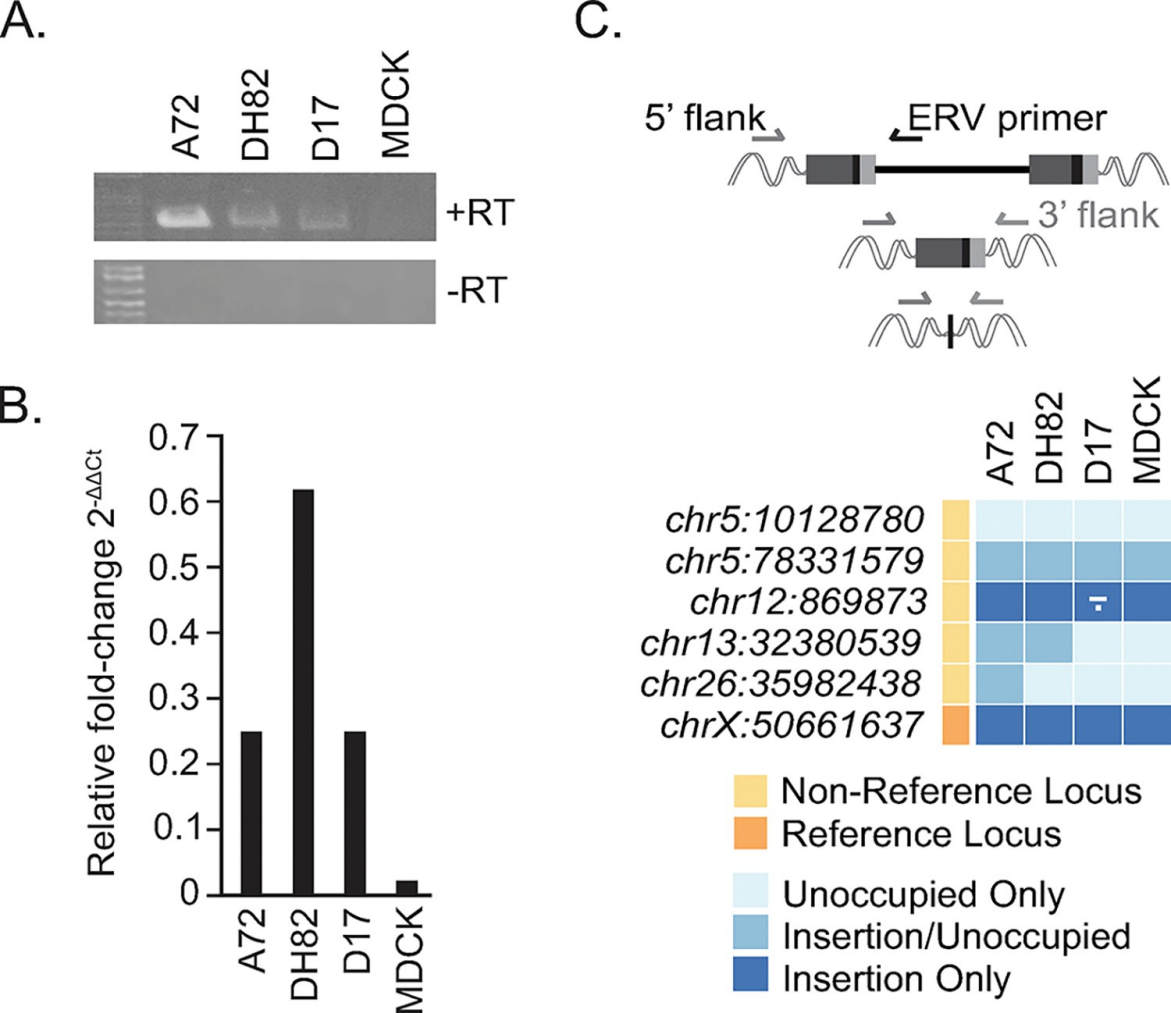
Expression of Fc1(a) *env* RNAs in canine cell lines. (A) Expression of *env* assessed by PCR of cDNA synthesized from total RNAs isolated from each cell line and visualized by gel electrophoresis. Reactions are shown for cDNA reactions performed with reverse transcriptase (+RT) and without (-RT) as controls. (B) Quantitative PCR of relative expression of the *env* calculated as mean fold-difference compared to endogenous control (GAPDH). The relative expression for each tissue sample was analyzed using the 2^-ΔΔCt^ method and the average shown for each. (C) Strategy for allele detection from isolated genomic DNA. Genotyping for *env* presence of the Fc1(a) loci confirmed to have an *env*-present provirus allele was performed via a two-step PCR screening on isolated genomic DNA. Primers were used to amplify occupied (*i*.*e*., full-length insertion, solo-LTR) or unoccupied (*i*.*e*., empty site) alleles. Dark blue indicates the presence of insertion alleles for loci on autosomes or the chrX pseudoautosomal region (X-PAR) [38]; mid blue indicates heterozygous representation; light blue indicates absence of the insertion only. For insertion presence of sites with variable insertion alleles, a dash indicates a full-length copy is present and a dot is indicative of a solo-LTR. All other provirus sites for which an insertion was detected reflect the presence of the full-length allele.

Gel electrophoresis of PCR amplicons with the *env*-directed primers confirmed expressed Fc1(a) in cDNAs synthesized from total RNAs isolated from the A72, DH82, and D17 cell lines (Fig 2A). The products were confirmed by Sanger sequencing. Despite multiple attempts, *env* amplicons were not visible by gel electrophoresis from MDCK cDNA. To further characterize *env* transcripts present in cell line cDNAs, we performed quantitative PCR (qPCR) using primers directed to the Fc1(a) *env* gene sequence and GAPDH as an endogenous control, with each sample averaged over triplicate runs (Fig 2B). Comparative CT values for *env* transcripts were detected at moderate levels from DH82 cells (2^-ΔΔCt^ of 0.63-fold), followed by A72 and D17 cells (2^-ΔΔCt^ of 0.254-fold and 0.249-fold, respectively). Consistent with PCR attempts, quantitative levels of *env* present in MDCK cDNA were several-fold decreased compared to the tumor-derived cell lines (2^-ΔΔCt^ of 0.036-fold).

Due to the high number of insertionally polymorphic loci known to be present in domestic dogs [21], the observed differences in *env* expression could possibly be explained by variability in presence of Fc1(a) proviruses in each cell line. To discriminate such possible source proviruses contributing to expression, we genotyped the allelic presence of *env*-possessing proviruses from genomic DNA isolated from each cell line. For these purposes, two PCRs were run per locus. The first PCR included a locus-specific primer designed to target the flanking genomic region that was paired with an Fc1(a)-specific internal primer to infer the presence of a full-length insertion; a second PCR included primers flanking each LTR-genome junction to detect the presence of the solo-LTR or unoccupied allele. Representative products for each insertion were sequenced and aligned to the CanFam3.1 genome build to confirm the correct amplified products and flanking genomic region for each locus. The obtained genotypes were tabulated and plotted for assessment (Fig 2C). As anticipated, the chrX:50,661,637 provirus was detected as a provirus in all cell lines, and none possessed the unoccupied allele or solo LTR at that locus. The remaining insertions were variably present, with the highest representation in the A72 cell line (another five loci had at least one *env*-possessing provirus present), followed by DH82 (another four loci), and D17 and MDCK (another three loci). Of note, the genotypes obtained for D17 and MDCK were in similar agreement, the only difference being the chr12:869,873 locus genotyped as provirus/solo-LTR in D17 that was present in homozygous state in MDCK. Given the similarity in *env* presence between the two cell lines, this latter observation suggests differences in *env* expression due to cell line-specific modulations. Also, as immortalized cells there are likely chromosomal aberrations or other alterations present that could consequently influence Fc1(a) expression, for example effects to gene regulation such as genomic methylation patterns or varying rates of cellular division [26].

### Fc1(a) *env* RNAs are expressed in healthy adult canine tissues

**Fig 3:**
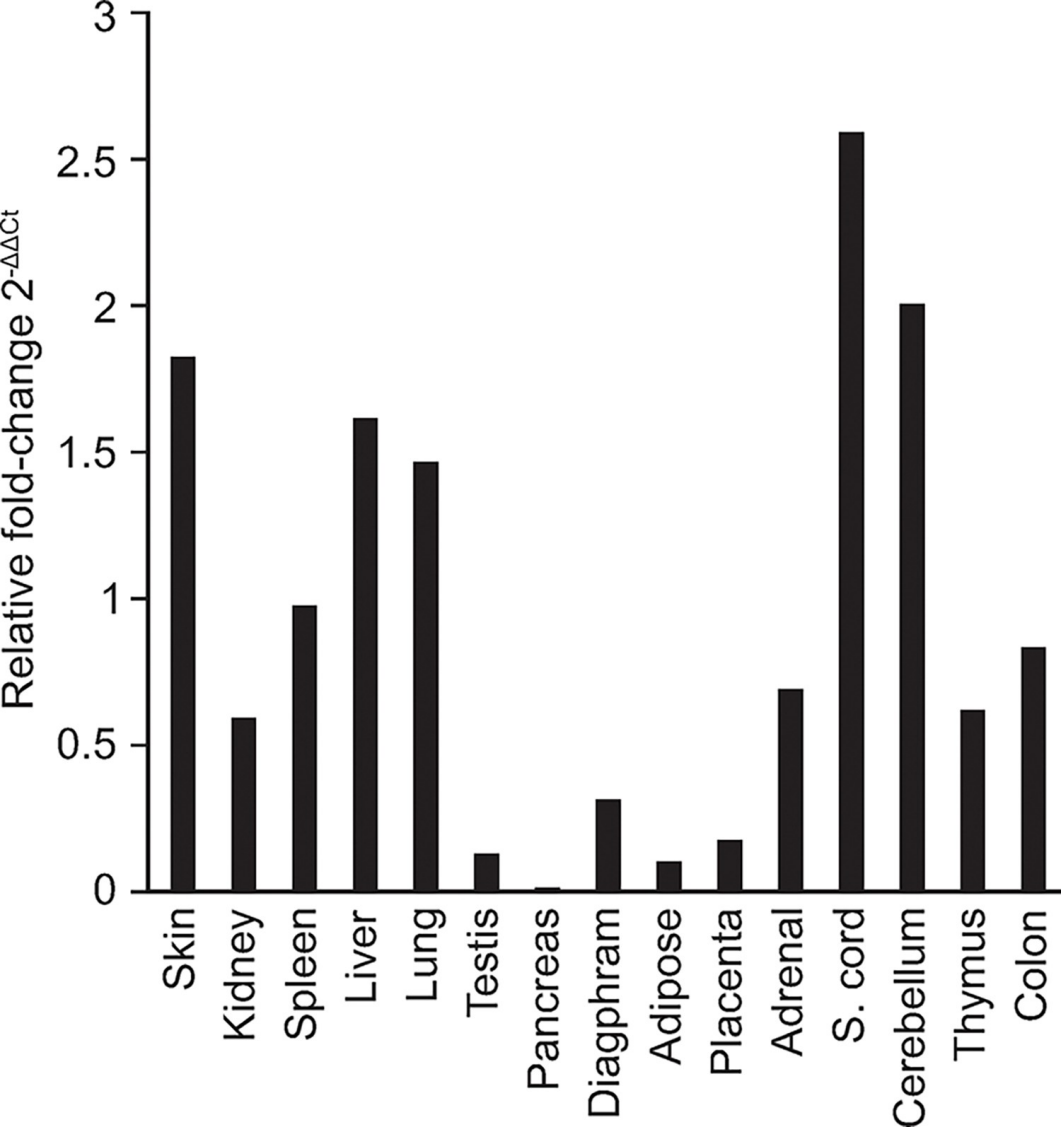
Fc1(a) *env* expression in tissues from healthy dogs. Samples were purchased from the biobank Zyagen, Inc., and consisted of a panel of cDNAs obtained from a total of 15 beagle dog tissues. The expression of the Fc1(a) *env* gene in tissues from healthy beagle dogs was assessed by quantitative PCR. Fold changes were calculated using the 2^-ΔΔCt^ method.

The expression of several ERV-derived lineages in humans and non-human animal models has been shown to differ between tissues associated with disease or cancers, as well as within tissues corresponding to normal physiological states. Given our detection of Fc1(a) *env* transcripts in cultured cell lines, we next examined *env* expression in tissues from healthy dogs using the same approach. To assess putative patterns of *in vivo* transcription of *env*, we performed qPCRs of Fc1(a) *env* in cDNAs synthesized from a panel of 14 tissue types from adult beagle dogs. Individual qPCRs were run for each tissue type targeting *env* and the obtained values calibrated using the corresponding GAPDH values as an internal control. We observed variable levels of *env* across tissue types, with relative expression varying from 2^-ΔΔCt^ of 0.006-fold (pancreas) to 2^-ΔΔCt^ of 2.63-fold (spinal cord) (Fig 3). Between the two there was wide variability in *env* expression across tissue types relative to GAPDH, with two tissue types over a difference of 2-fold (spinal cord, cerebellum). Collectively these results confirm the Fc1(a) *env* is expressed within the total RNAs of canine tissues and suggest Fc1(a) promoters may be active in distinct tissue types.

### Fc1(a) *env* expression tends to be elevated in canine tumors

In principle, because younger ERV integrants result from germline invasion of a relatively recently infectious source, they are likely candidates to have retained the potential to be biologically active. Previously, transcripts, retroviral enzymes and other products were characterized in cancer-associated tissues sampled from dogs with lymphoma or leukemia or severe immunosuppression [7–15]. We reasoned a possible contributing source of these observations could be due to expressed Fc1(a) insertions given the recent germline colonization and intactness of the group. We therefore expanded our study to measure Fc1(a) expressed *env* levels in tumors sampled from 19 dogs with various cancer diagnosis (S2 Table).

**Fig 4:**
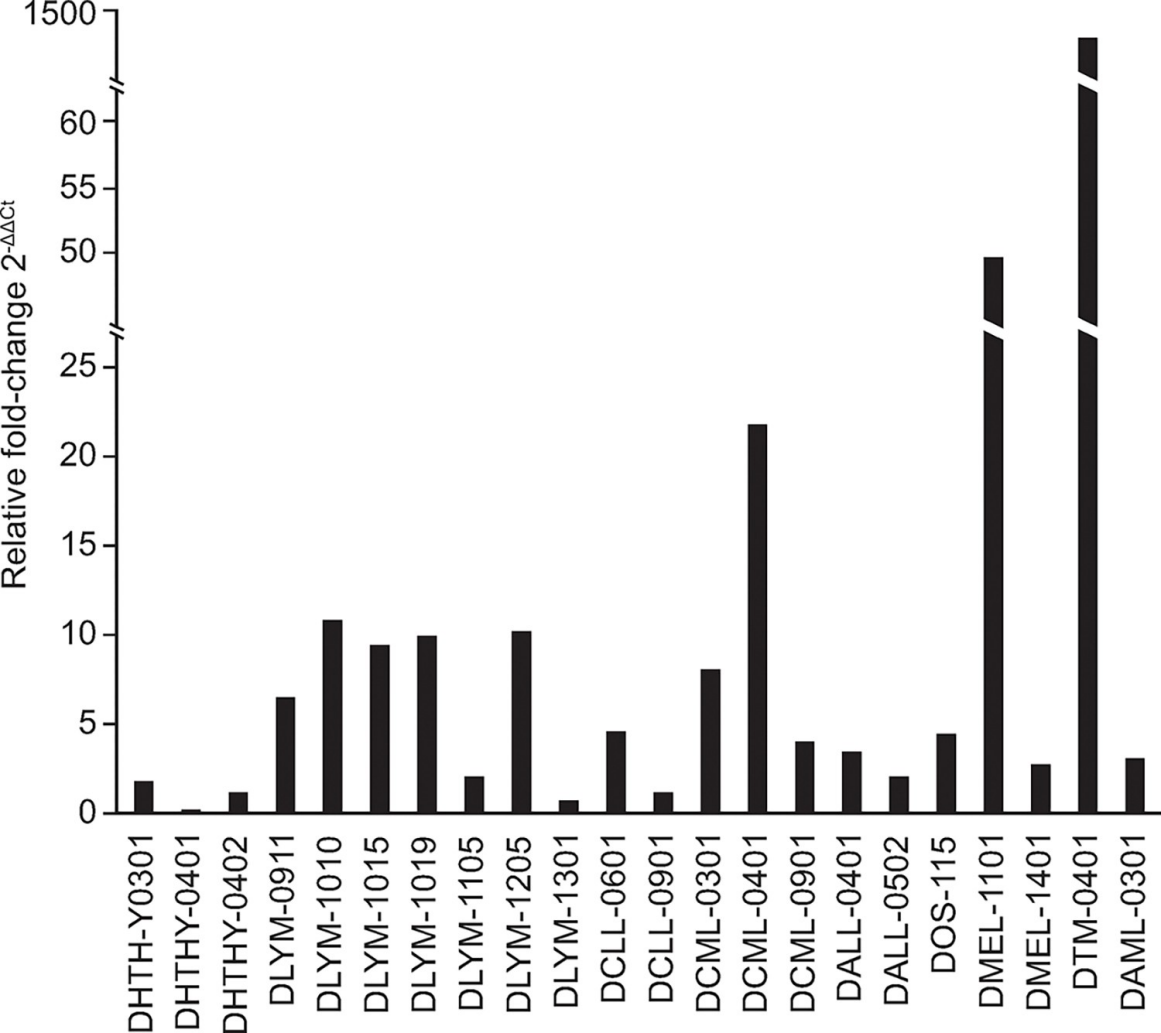
Expression of Fc1(a) *env* RNAs in tumors from dogs with cancer. Expression of the Fc1(a) *env* gene was evaluated by quantitative PCR in diseased tissues from dogs with cancer and as well as tissues from healthy animals. RNA was isolated and used to synthesize cDNA from each sample and relative expression of *env* subsequently analyzed in using the 2^-ΔΔCt^ method. Sample names are indicated according to clinical group. Samples are from healthy animals (DHTHY, n = 3) and tumors of lymphoma (DYLM, n = 7), leukemias including chronic lymphocytic leukemia (DCLL, n = 2), chronic myelomonocytic leukemia (DCML, n = 3), acute lymphocytic leukemia (DALL, n = 2), and acute myelomonocytic leukemia (DAML, n = 1), melanoma (DMEL, n = 2), osteosarcoma (DOS, n = 1; DTM, n = 1).

**Fig 5:**
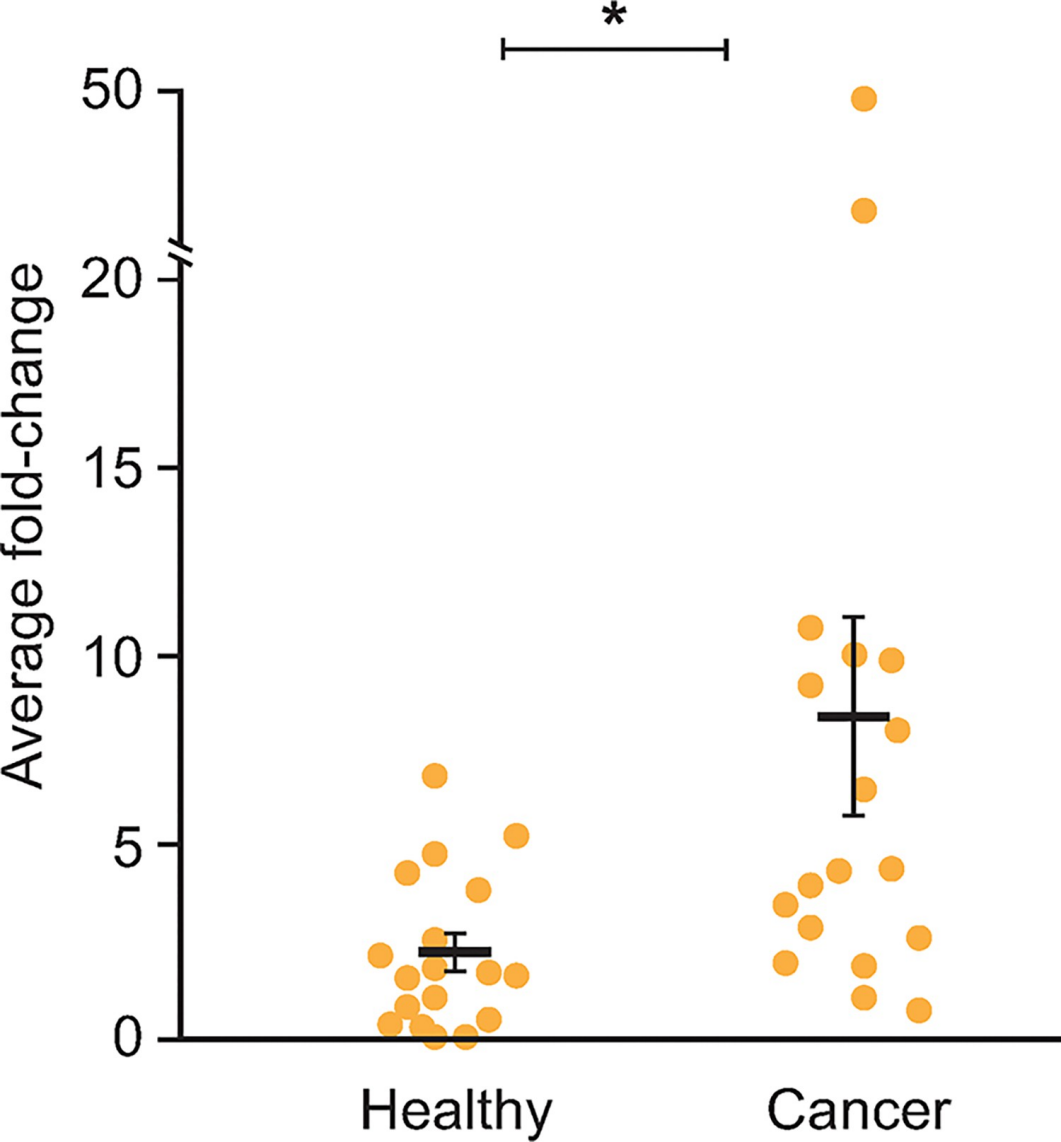
Fc1(a) *env* expression in healthy and tumor tissues. Comparison of *env* expression between healthy samples and diseased samples. Each dot represents the individual sample average fold-difference value corresponding to total healthy (adult healthy tissues as well as DHTHY samples; n = 18) or disease samples (n = 19). For both the healthy and diseased groups, the overall average (black bar) and standard error is shown. The * symbol signifies a statistically significant difference (*p*>0.02) between sample groups as calculated by an unpaired *t*-test.

Our analysis confirmed that cDNAs corresponding to *env* were detectable at variable levels across all samples (Fig 4). Expression in blood samples from healthy dogs ranged from 2^-ΔΔCt^ of 0.53-fold (DHTHY-1401) to 1.38-fold (DHTHY-0301) relative to the endogenous control. In contrast, relative *env* levels in tumor samples were highly variable and tended to be elevated compared to levels from healthy samples. For example, increased fold-differences in expression were observed in 15 of the 19 tumor samples compared to the average of healthy blood samples. Nine of 19 tumor samples were over 5-fold increased relative to background and six of those nine were >10-fold increased. Most of these samples were from lymphoma affected tissues, though it should be noted these were the most abundant cancer type examined (seven of the 19). The highest increased fold differences in *env* expression were observed in samples from dogs diagnosed with chronic myelomonocytic leukemia, melanoma, and osteosarcoma, with 2^−ΔΔCt^ values of 22.02-fold (DCML-0401), 48.51-fold (DMEL-1101), and 1,402.29-fold (DTM-1401), respectively. Treating the lattermost fold expression from DTM-1401 as an outlier and excluding it from further calculations, the overall average *env* expression within tumor samples was 2^-ΔΔCt^ of 8.53-fold. The relative expression of *env* was significantly higher in the cancer samples than in the blood samples from healthy dogs using a *t*-test (*p*<0.002), again after excluding DTM-1401 (Fig 5). Collectively, these data indicate Fc1(a) *env* expression was elevated but variable in presence and relative level in malignant tissues compared to blood or other tissues from healthy dogs.

### Fc1(a) proviruses are insertionally polymorphic in healthy and tumor tissues of dogs

**Fig 6:**
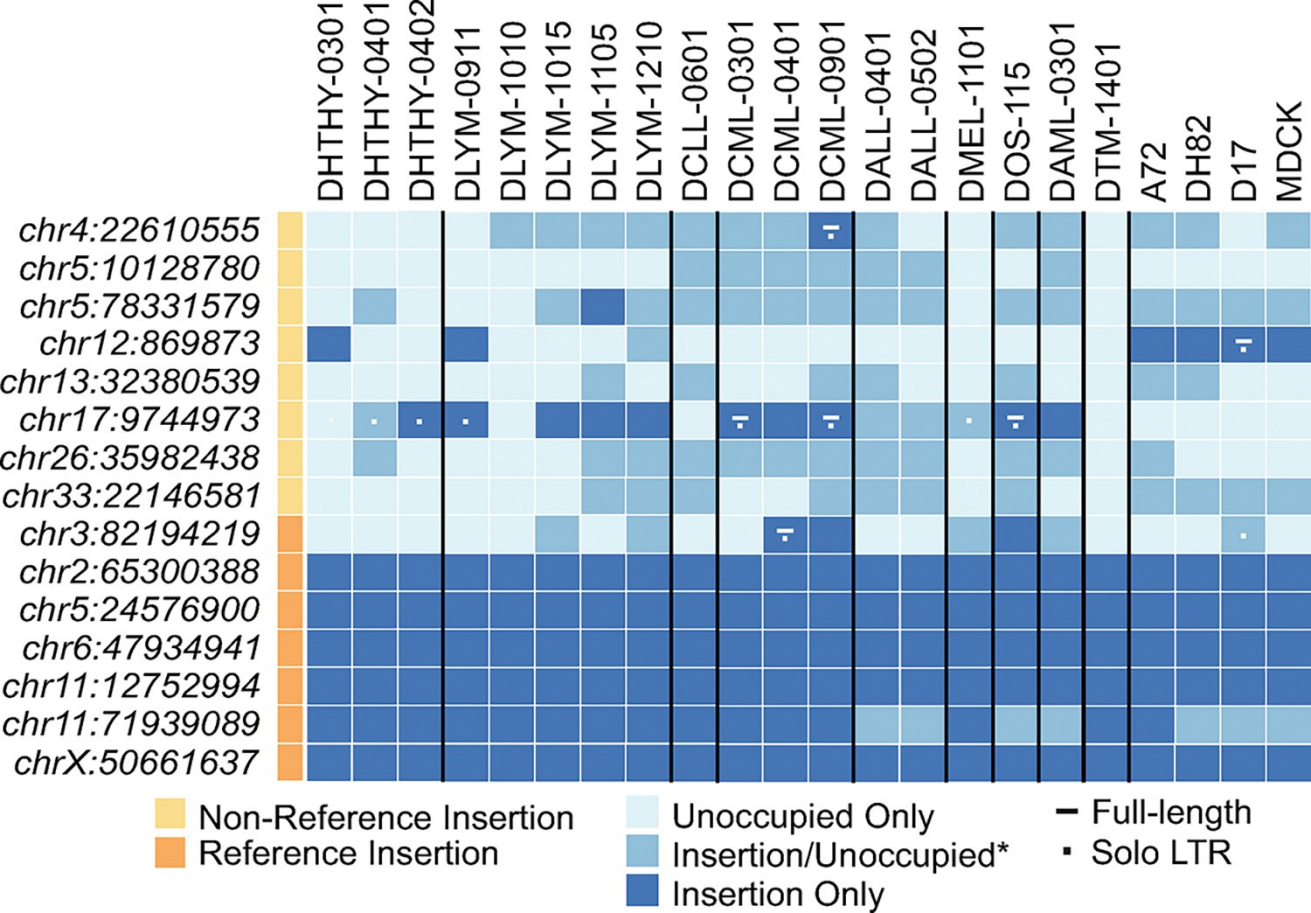
Fc1(a) provirus prevalence among cancer samples. Genotypes were obtained as in Fig 2C from genomic DNA isolated from 17 tissues corresponding to tissues from 14 tumors as well as blood from 3 healthy animals. Dark blue indicates two insertion alleles on autosomes or the X-PAR; mid-blue indicates heterozygous representation for the site on autosomes, *or an insertion allele on chrX outside the X-PAR in males; light blue indicates presence of the empty allele only. For insertion presence of sites with variably detected insertion alleles, a dash indicates a full-length copy and a dot the solo-LTR. All other provirus sites for which an insertion was detected reflect the presence of the full-length allele. At left, gold indicates absence of the insertion from CanFam3.1; orange indicates presence in CanFam3.1. Insertion coordinates are shown at left and correspond to the leftmost insertion breakpoint in CanFam3.1. Sample names are indicated at the top according to clinical group. Samples are from healthy animals (DHTHY, n = 3) and tumors from canines diagnosed with lymphoma (DYLM, n = 4), leukemias including chronic lymphocytic leukemia (DCLL, n = 1), chronic myelomonocytic leukemia (DCML, n = 3), acute lymphocytic leukemia (DALL, n = 2), and acute myelomonocytic leukemia (DAML, n = 1), melanoma (DMEL, n = 1), osteosarcoma (DOS, n = 1; DTM, n = 1).

The observed variability in *env* transcript levels among our sample set could be due to differential contributions of expression among insertionally polymorphic Fc1(a) loci. To assess whether the total candidate source Fc1(a) elements in each sample correlated with total *env* expression, we therefore sought to genotype the presence of the *env*-possessing full-length elements from genomic DNAs of the same samples, as well as extend the analysis to additional full-length elements to obtain a snapshot of prevalence across samples (S1 Table). We obtained genomic DNA from cryopreserved cells or tumor tissues from 13 of the 19 samples collected from dogs with cancer, as well as from blood from three healthy canines. These samples were utilized as sources for proviral screening, again using two PCRs per locus to genotype the presence of a proviral allele, solo-LTR, or unoccupied site for each Fc1(a) locus (refer to Fig 2C). Data corresponding to obtained genotypes (S1 Data) was visualized using a heat map to assess genotypes over the sample set (Fig 6).

Consistent with previous findings of Fc1(a) presence as detected in modern breeds [21], five of the 14 insertions were fixed as provirus alleles in all samples, one of which (the chrX:50,661,637 integrant) represented the only *Canis*-fixed provirus with a predicted *env* ORF. As expected, we observed variability in the presence/absence of the remaining nine Fc1(a) proviruses between samples, including the remaining loci with clear *env* reading frames, as well as variable frequencies across all samples screened. Most of the Fc1(a) loci were heterozygous for the provirus and empty alleles, with few instances of samples homozygous for the provirus insertion (*e*.*g*., chr12:869,873: DHTHY-0301, DLYM-0911), or samples with variable presence of the insertion allele and/or empty site (*e*.*g*., chr4:22,610,555: DCML-0901). Aside from the provirus on chrX, there were 10 samples with at least one additional provirus predicted to possess an *env* reading frame. All proviruses were present in at least one sample and considerable allele frequency variation was observed for the nine unfixed loci. No one sample possessed all seven Fc1(a) proviruses with putatively intact *env*. We note that while the presence of *env* RNAs in these samples can be accounted for by LTR activation, the possibility of their presence as part of a host mRNA is not excluded (see Discussion). Further insight into the transcriptional regulation of the Fc1(a) insertions will require more comprehensive analyses.

### Members of the Fc1(a) lineage exhibit high levels of insertional polymorphism in dogs

A provirus contains regulatory sequences for its own transcription within its LTRs. Since the LTR sequence is predicted to be preserved during recombination resulting in solo-LTR formation, the recombinant allele likewise maintains the same transcriptional potential. As such, LTRs have been shown to impact genomic function in humans and other animal models by altering local or long-range gene regulation via introduced promoter, enhancer, or other regulatory activities (recently reviewed in [27]). The genomic distribution of Fc1(a) includes members within or near genes and display varying allelic frequencies in the dataset [21], thus presenting the opportunity for a preliminary examination for putative relationships with disease or phenotype.

**Fig 7:**
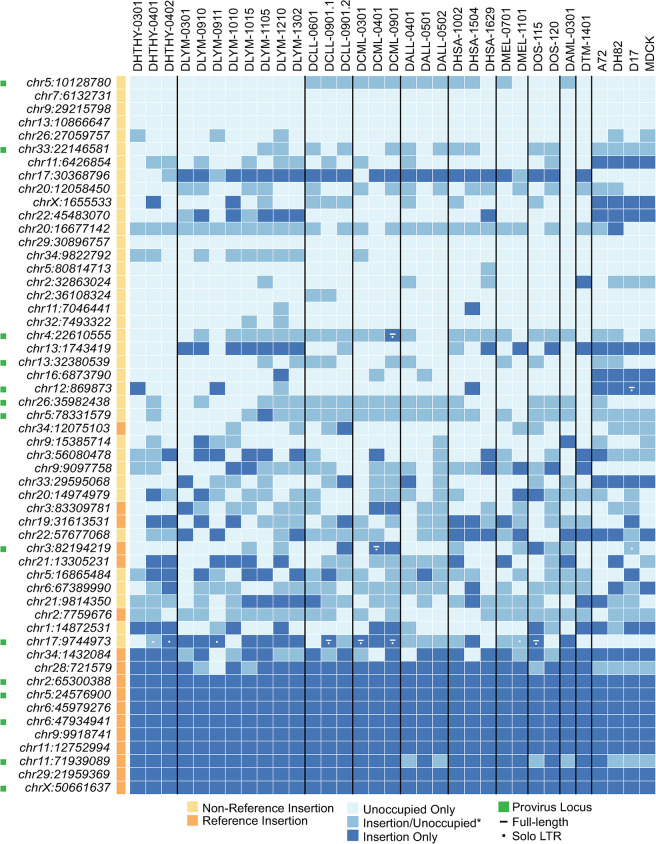
Insertional polymorphism of Fc1(a) insertions. Genotypes were obtained using the strategy as in Fig 2C for 54 Fc1(a) loci utilizing genomic DNA isolated from blood, canine tumor tissues, and cell lines A72, D17, DH82, MDCK. Sample names are indicated at the top according to clinical group. Samples were from healthy animals (DHTHY, n = 3) and tissues from canines diagnosed with lymphoma (DYLM, n = 8), chronic lymphocytic leukemia (DCLL, n = 3), chronic myelomonocytic leukemia (DCML, n = 3), acute lymphocytic leukemia (DALL, n = 3), acute myelomonocytic leukemia (DAML, n = 1), hemangiosarcoma (DHSA, n = 3), melanoma (DMEL, n = 2), osteosarcoma (DTM, n = 1; DOS, n = 2). At left, gold indicates ‘non-reference’, or absence of the insertion from CanFam3.1; orange indicates ‘reference’ insertion, or presence in CanFam3.1. Dark blue indicates insertion alleles on autosomes or the X-PAR; mid blue indicates heterozygous representation for the site on autosomes, *or an insertion at a chrX locus outside the X-PAR in males; light blue indicates absence of the insertion. For insertion presence of sites with variably detected insertion alleles, a dash indicates a full-length copy and a dot the solo-LTR. All other provirus sites for which an insertion was detected reflect the presence of the full-length allele. Genotypes are shown for sites shown to have a full-length copy of the insertion allele (indicated by green squares) or solo-LTR only in addition to the empty, or unoccupied allele. Insertion coordinates are shown at left and correspond to the leftmost insertion breakpoint in CanFam3.1.

Of the Fc1(a) 157 insertions currently annotated in *Canis spp*., 145 insertions were previously deemed as having resolvable integration sites and were genotyped in 332 genomes of dogs and wild canids, including 137 modern breeds [21]. In that study, genotypes were inferred *in silico* by re-mapping Illumina reads over recreated alleles corresponding to the insertion or unoccupied states, permitting estimates of prevalence for individual Fc1(a) insertions. Occupied alleles for a total of 40 insertionally polymorphic Fc1(a) loci were detected in the genomes of modern breeds (Fig 1), with allele frequencies ranging from less than 0.01 (single copy detected in 137 dogs) to 0.85 [21]. We examined these 40 Fc1(a) insertions in an expanded PCR-based genotyping analysis to assess the distribution of Fc1(a) alleles across samples in our present study, as well as draw comparison to previous estimates among modern breeds reasoning the comparison was most representative of expectations. We added a subset of loci previously deemed as either absent from or fixed in modern breeds by *in silico* genotyping or that possessed a provirus allele; this resulted in a total of 54 sites examined (S1 Table). For screening, genomic DNA from tumors of an additional 13 diagnoses were included for a total of 29 samples examined (S2 Table), thus providing an extended view of Fc1(a) genotypes among samples from dogs with distinct cancers. Raw genotypes (S1 Data) were scored and plotted for assessment after ordering by inferred *in silico* allele frequencies for each Fc1(a) locus (Fig 7).

The overall frequencies observed for each Fc1(a) locus were generally consistent with previous *in silico* estimates [21]. For example, three annotated Fc1(a) that were previously identified in wild *Canis spp*. only (*e*.*g*., wolves, coyotes, and jackals; orange squares in Fig 1) were likewise absent in our screens (*e*.*g*., chr7:6,132,731, chr9:29,215,798, chr13:10,866,647). Also consistent with previous findings were eight sites fixed among *Canis spp*., with exception of a solo-LTR (chr11:71,939,089) which was heterozygous in four of the samples. As with our initial screens, the 14 sites confirmed to have a provirus allele were variably present and no individual possessed all seven Fc1(a) proviruses with a putatively intact *env*. Treating either insertion allele as presence (*i*.*e*., provirus, solo-LTR) and the unoccupied as absent (*i*.*e*., empty), overall allele frequencies ranged from 0.017 (chr5:80,814,713, single copy) to 0.91 (chr28:721,579) across the loci genotyped. Thus, the majority of Fc1(a) insertions were detected in frequencies consistent with expectations [21]. Some putative trends were observed by genotyped presence corresponding to sample group as well as deviation from expectations as inferred from previous estimates in modern breed individuals. Among putative deviations were two proviruses not detected in our previous study, but that were genotyped here as heterozygous (provirus, empty) in multiple samples: chr5:10,128,780 and chr33:22,146,581. Inconsistencies with previous estimates were also observed for loci having presence or absence of a solo-LTR, examples include but are not limited to chr17:30,368,796, chr22:45,483,070, chr20:16,677,142 (also refer to Fig 7; see Discussion). No additional locus was found to possess a provirus allele among the genotyped samples.

Our genotyping strategy was two-fold in that it permitted a preliminary assessment of locus prevalence between groups as well as examination of the placement of Fc1(a) loci within or near gene locales in the clinical context. A relationship may be drawn by the genetic association of the presence of an inherited provirus or solo-LTR to a particular phenotype or disease state, as has been characterized in other animals, for example in mice [28]. Given the observed variability in presence, high levels of sequence similarity, and putative functional conservation, the Fc1(a) lineage presents candidates for such a scenario in the domestic dog. Excluding loci fixed in all samples, of the polymorphic insertions present in modern breeds, eight are located within introns of predicted dog gene models. From a predicted gene model start, another two, six, and six are present within 5kb, 10kb, and 25kb, respectively (S1 Table; also see Discussion). Though preliminary, these data collectively show that ERV-derived elements, including proviruses that we find to be capable of contributing to biological expression, have impacted structural genome variation in dogs and provide an unexplored means to examine potential genetic links with disease states in this species.

## Discussion

ERVs have the potential to generate viral products, or to alter normal host gene expression through the contribution of a promoter sequence from an LTR. In this regard, lineages having ‘young’ members are of interest given inferred promoter activities as well as presence within new genomic contexts. With few exceptions (for example, see [29,30] and as discussed below), in comparison to other animals ERV biological activities in dogs have been relatively understudied and there is a deep gap in our understanding of such processes in this model species. We previously characterized the emergence and expansion patterns of the γ-like Fc1(a) ERV lineage revealing numerous loci with insertional polymorphism in extant Canidae including domestic dogs [21]. An inferred Fc1(a) progenitor was intact, and sequence comparisons revealed that the youngest proviruses should require few mutations to restore the lineage consensus. Expression from such relatively recent ERV integrants has been linked to disease in humans and other animals (reviewed in [1,31]). In this regard, expression of Fc1(a) members would be consistent with activation contributing to, at least in part, previous reports of γ-like retroviral products in tissues of canine cancers, thus motivating our study. Our findings provide a precedent of further examination of the biological impact and co-evolutionary dynamics of between retrovirus and host in the underexplored system of the dog.

The *env* gene is most frequently present among young Fc1(a) proviruses with seven possessing an uninterrupted ORF of which a majority are without obvious changes that would alter function [21]. We examined *env* expression in cDNA synthesized from canine cell lines derived from various sources. While this approach was thus focused on proviruses that possess an *env* gene (S2 Fig), the possibility of expression from proviruses with an *env*-disrupting deletion is not excluded (refer to S1 Table). Moderate levels of *env* transcripts were detected in cell lines, of which MDCK consistently had the lowest level of expressed *env* observed. To interrogate the presence of source candidates, we genotyped Fc1(a) proviruses predicted to possess an *env* ORF using genomic DNA from each cell line. Multiple *env*-possessing Fc1(a) proviruses were confirmed as present and genotypes varied between samples. A72 and DH82 possessed proviruses at six and five loci, respectively, and D17 and MDCK shared the same four. Particularly given the similarity in provirus content between these latter two cell lines, we interpret the differences in *env* expression to indicate differences in contributing factors to the relative *env* transcript levels. For example, inherent differences in the tissue type from which each line was derived, increased proliferation with shortened cell cycle times, possible genomic rearrangement, or malignant phenotype. The potential of contribution of *env* transcripts from not-yet annotated copies cannot be ruled out. Expanding our analysis to examine Fc1(a) *env* expressed in healthy adult dog tissues revealed variable levels of expression across tissue types, with increased fold differences in tissues from spinal cord, cerebellum, skin, liver, and lung. The highest increased fold differences of expressed *env* in our analysis were observed in neurological-related tissues of the spinal cord and cerebellum. Though the consequence is unclear, elevated levels of ERV expression in the nervous system has been reported in other animals [32,33].

Deregulation of ERV proviruses is well-known to occur in cancers and other disease states in humans and other animals [1]. To examine whether similar patterns would be observed for Fc1(a) proviruses, we analyzed Fc1(a) *env* presence in total RNAs of healthy tissues as well as tumor samples of diagnosed cancers in canines. We observed variable patterns of expression that tended to be elevated in tumors (*p*<0.02). Five of the seven lymphomas, the most represented cancer examined, had increased *env* of 5- to 10-fold difference compared to cDNAs of healthy tissues. Greater fold differences in *env* expression were respectively from a metastatic melanoma (DMEL-1101) and chronic myelomonocytic leukemia (DCML-0401). The latter sample DCML-0401 had increased *env* relative to other samples from shared diagnosis and was in blast crisis; this most progressed phase is typically accompanied by cell fusions and large scale chromosomal aberrations, thus likely impacting gene regulation [26,34]. The highest increased fold difference of *env* expression was measured from an osteoblastic giant cell tumor (DTM-1401). Genotyping inferred candidate sources of at least one Fc1(a) provirus with a predicted *env* ORF for each sample. Though there was variability in the presence of insertionally polymorphic Fc1(a) proviruses, the genotyped DCML samples shared three such *env*-possessing proviruses (also see below).

Our findings are supported in part by a previous study by Cornelis *et al*., [29] in which *env* ORFs, including from Fc-related and unrelated ERV groups, were assessed for expression as candidate genes for a carnivore syncytin, leading to the identification of an ERV-R *env* derived gene, ‘syncytin-Car-1’, for roles in placentation. In that study, variable expression between distinct tissue types was observed in quantitative RT-PCRs over a similar composite tissue panel including the candidate chrX:50,661,637 Fc1(a) *env* (*’canis-env3*’ in that report). An *in silico* examination of the primers used in that study suggests specificity to the chrX *env* target alone, such that other Fc1(a) proviruses were most likely excluded from detection. We suggest expression of the chrX provirus may thus contribute, at least in part, to the Fc1(a) *env* transcripts we observe in tissues. Other support for Fc1(a) expression comes from the recent mapping of CfERV cDNAs of oral malignant melanoma (OMM) transcriptomes, in which Kitao *et al*. identified source contributing proviruses [30]. Subsets of these OMM transcripts mapped to Fc1(a) internal sequences, including the abovementioned chrX *env*, the chr11:12,752,994 *gag* region (premature stop predicted in CanFam3.1) and *pol* ORF, as well as the chr3:82,194,219 *gag* and *env* regions (also premature stops). As mapping of cDNAs was limited to proviruses present in CanFam builds [30], any contribution to total OMM RNAs from non-reference proviruses was not further explored.

While the presence of Fc1(a) RNAs both here and elsewhere are presumed to be at least in part accounted for by activation of LTR promoter functions, functional analyses of promoter activities of CfERV LTRs in general is currently lacking, and the possibility of intronic transcription as part of a host gene for at least some ERV loci is not excluded. In this regard, one provirus that has predicted *pol* and *env* ORFs (chr26:35,982,438; one of four Fc1(a) with both *pol* and *env* ORFs) sits within the dog gene model for *PRKG1* (also discussed below). This gene is predicted to encode three isoforms of cyclic GMP-dependent protein kinase with roles in signal transduction. The provirus was genotyped as present in a subset of samples in heterozygous state (*i*.*e*., provirus/empty), thus offering a candidate for such a scenario of passive expression. Additional Fc1(a) solo-LTR insertions within genes include, but are not limited to, homologs involved in tumor suppression (*EXT1*) and immune functions (*WDFY4*) (S1 Table). Further insight into the transcriptional regulation and functional context of the Fc1(a) insertions, for example by the presence of unspliced or spliced transcripts and pinpointing all source loci will require more comprehensive analyses based on expression. Functional analyses in this context are ongoing.

As LTRs are known to contain regulatory sequences for host transcriptional machinery, gene-proximal insertionally polymorphic Fc1(a) loci lend candidacy for alteration of normal gene regulation. For example, the abovementioned chr26:35,982,438 provirus. The insertion is present in 17 of the 26 samples from dogs with cancer and its allelic presence is consistent with the detection of proviruses genotyped at chr5:10,128,780 and chr5:78,331,579 among all samples analyzed from chronic myelomonocytic, chronic lymphocytic, or acute lymphocytic leukemias (DCML, DCLL, and DALL, respectively), indicating possible trends in Fc1(a) presence per sample group. A similar example, among others, is the chr17:30,368,769 solo-LTR that was conspicuously present by genotyping among all but two tumor samples in predominantly homozygous states, though the significance of this observation is unclear. Other solo-LTRs are proximal to genes with functions involved in gene regulation (*EED*, *BUD13*, *TCF19*), cytoskeletal dynamics (*PLEKHG4*), and others (S1 Table). In this regard, understanding the properties of Fc1(a) LTRs as transcriptional promoters will not only shed light on functions as drivers of proviral expression but also impact to genes located nearby, particularly given their conservation in sequence between loci. The high levels of insertional polymorphism of this young ERV lineage are highly suggestive of a potential for biological impact. We thus present a preliminary view of the Fc1(a) landscape in a disease context and offer justification for future focused analysis.

In summary, our study confirms expression of Fc1(a) *env* in canine tissues and finds this expression is increased in tumors. We suggest the transcriptional deregulation of this ERV lineage is responsible, at least in part, for previous observations of γ-like retroviral products in cancers. By genotyping, we link these findings to candidate source loci. Several proviruses are predicted to have one or more ORFs, raising the possibility of expressed products that retain putative function. Though additional analyses are necessary to begin to draw any biological association or impact of Fc1(a) presence in this species, these findings raise questions of the relationship of this expression therein, mechanisms of activation, as well as the potential for formation of infectious virus. Though no Fc1(a) provirus identified thus far is predicted to possess ORFs for all genes, sequence analysis implies very few changes would be required to restore intactness and this scenario could, based on our preliminary analysis of existing loci, be resolved via template switch of co-packaged viral RNAs sourced from distinct loci. Indeed, the Fc1(a) progenitor itself arose as a viral recombinant from distinct source ERV groups that placed an RDR-group *env* in the infecting virus. Its subsequent spread in dog ancestors also resulted in multiple unique recombinant proviruses now present in the dog ERV fossil record. The possibility that rare but intact proviruses are presently segregating in dogs or related extant canids remains.

## Materials and methods

### Ethics statement

All of the samples from dogs with cancer that were used for this study were obtained as part of medically necessary diagnostic procedures with written consent of the owners. No animals were harmed in the process of obtaining samples and in all cases, the disease occurred naturally and was not induced or in any way accelerated by the diagnostic procedures. The samples were collected over a period spanning more than 20 years at three institutions including the American Medical Center (AMC) Cancer Research Center, the University of Colorado, and the University of Minnesota, with approved protocols that were renewed every three years through the AMC Cancer Research Center Institutional Review Board (IRB), the AMC Cancer Research Center Institutional Animal Care and Use Committee (IACUC), the Colorado Multiple IRB (COMIRB), and the University of Minnesota IACUC. Oversight protocols included AMC 635040202, AMC 200201jm, AMC 2002141jm, 02905603(01)1F, and COMIRB 06–1008, approved by the AMC Cancer Research Center and the University of Colorado Institutional Review Board and Institutional Animal Care and Use Committees and protocols 0802A27363, 1101A94713, 1110A06186, 1312-31131A, 1507-32804A, and 1702-34548A, approved by the University of Minnesota Animal Care and Use Committee.

### Primer design for *env* detection

A nucleotide alignment was generated and manually edited in BioEdit [35] for 19 full-length Fc1(a) proviruses and the Fc1(a) consensus [21]. In total the alignment included 11 proviruses in the CanFam3.1 reference genome assembly and eight non-reference proviruses. Primers were then designed for qPCR of expressed transcripts to target a ~300bp segment within the *env* SU domain 6059–6357 bp from the Fc1(a)_CON_ start: Forward, 5’ CATGCCCAGAACTTGTTACTCA; Reverse, 5’ TGGTGGGAACTTTCTTGATG (S2 Fig). The targeted region is predicted to be present only in Fc1(a) proviral integrants that possessed the full-length *env* segment (a potential nine of the 19 annotated insertions, two of which possess inactivating premature stops: chr3:82,194,219, chr6:47,934,941; also refer to S1 Table). The primer products were assessed for target specificity using the *in-silico* PCR tool (https://genome.ucsc.edu/) against the CanFam3.1 assembly, which confirmed return of the three reference proviruses with a full length *env* segment. This step allowed us to both predict the size of the amplified products and assess specificity of the primer sets to the Fc1(a) loci.

### Cell lines

Canine cell lines were purchased from the American Type Culture Collection (ATCC) and were derived from tumor and non-tumor tissue types. Tumor-derived lines included A72 unknown tumor type, DH82 malignant histiocytosis, D17 osteosarcoma; non-tumor derived was MDCK epithelial. Cells and culture conditions were maintained as follows. A72 were maintained in Leibovitz media (ATCC) supplemented with 10% fetal bovine serum (FBS; Gibco) and 20 U/mL Penicillin/Streptomycin (Gibco), DH82 were maintained in Eagle’s minimal essential medium (EMEM; ATCC) with 15% heat-inactivated FBS and 220 U/mL Penicillin/Streptomycin, and D17 and MDCK were maintained in EMEM with 10% FBS and 20 U/mL Penicillin/Streptomycin. D17, DH82, and MDCK were grown at 37°C in 5% CO_2_; A72 were grown at 37°C in 0% CO_2_. For passaging, cells were grown to ~80% confluency and washed in 1x PBS (Gibco), harvested in 0.25% Trypsin-EDTA (Gibco), and subcultured in a 1:4 ratio in complete media.

### Canine tissue samples

Expression of *env* in healthy tissues was analyzed using purchased first-strand cDNAs from adult beagle dogs (Zyagen, San Diego, CA). cDNAs were from 15 tissues including skin, kidney, spleen, liver, lung, testis, pancreas, diaphragm, adipose, placenta, adrenal, spinal cord, cerebellum, thymus, and colon. A total of 29 samples were collected from spontaneous tumors of companion dogs. Samples were from lymphoma (DLYM, n = 10), melanoma (DMEL, n = 3), osteosarcoma (DTM, n = 1; DOS, n = 2), chronic lymphocytic leukemia (DCCL, n = 3), chronic myelomonocytic leukemia (DCML, n = 3), acute lymphocytic leukemia (DALL, n = 3), acute myelomonocytic leukemia (DAML, n = 1), and hemangiosarcoma (DHSA, n = 3). Three additional blood samples were obtained from healthy dogs (DHTHY, n = 3). Demographic information pertaining to the dogs from which these samples were obtained is summarized in S2 Table.

### RNA extraction

For obtaining RNA from cell lines, cultured monolayers were initially harvested in 0.25% Trypsin-EDTA (Gibco) and washed twice in 1xPBS with centrifugation at 1,000 rpm for 4 minutes (mins). For obtaining RNA from tissue samples, the tumors were sectioned and homogenized prior to lysis; for cryopreserved cells, 500 μl of each sample was utilized. RNA extraction was performed for all sources using a NucleoZol RNA extraction kit (Machery-Nagel) according to the manufacturer’s protocols. Briefly, each cell pellet was homogenized by vortexing in 500 μL NucleoZol and 200 μL RNA free water and the mixture was then centrifuged 15 mins at 12,000g following a 15 mins incubation at room temperature. The supernatant was collected, from which RNA was purified using the NucleoSpin RNA Set for NucleoZol (Machery-Nagel) according to the manufacturer’s instructions. Complete removal of DNA was verified through GAPDH PCR of templates consisting of extracted RNA and synthesized cDNA for all samples. All remaining RNA was stored at -80°C.

### cDNA synthesis

RNA was eluted from a NucleoSpin RNA column in 60 μL RNAse-free water and immediately subjected to a reverse transcription using random hexamer primers with the M-MuLV Reverse Transcriptase kit (New England Biolabs) following the manufacturer’s protocols. All remaining RNA was stored at -80°C and the reverse transcribed cDNA stored at -20°C. A *Taq* PCR reaction was run on the extracted RNA and reverse transcribed cDNA from each sample using previously published GAPDH primers [36] to confirm the quality of cDNA following reverse transcription and the absence of genomic DNA in the extracted RNA samples. PCR reactions were performed using 0.125 U *Taq* polymerase (Invitrogen) in 10x buffer, 2.5 μM dNTPs, 10 μM either primer, and 2.5 μM MgCl_2_ under the conditions of initial denaturation at 95°C for 2 mins followed by 35 cycles of 95°C for 30 secs, 59°C for 30 secs, 72°C for 1:15 mins, and a final extension at 72°C for 3 mins. 10 μL of the PCR reaction was visually assessed by electrophoreses in 1% agarose and 1xTBE.

### PCR amplification

Synthesized cDNA was utilized as a template in PCRs to detect *env* expressed segments. PCR reactions were run with 1 μL cDNA in 10x buffer, 2.5 μM dNTPs, 10 μM each primer (as described above), 2.5 μM MgCl_2_, and 0.125 U *Taq* polymerase (Invitrogen). Reactions were performed in an Eppendorf Mastercyler under conditions of initial denaturation at 95°C for 2 mins followed by 35 cycles of 95°C for 30 secs, 59°C for 30 secs, and 72°C for 1:15 mins with a final extension for 3 mins at 72°C. Amplified products were assessed by gel electrophoresis in 1% agarose in 1xTBE to confirm presence and amplicon size. The remaining products were purified using a Nucleospin Gel and PCR Clean-up Kit (Machery-Nagel) according to the manufacturer’s protocol. Yield was assessed using a Nanodrop Lite (ThermoFisher).

### Quantitative PCR

cDNA products successfully amplified from nucleic acids from cell lines, tumor tissues, and lymph tissues were subjected to a quantitative PCR (qPCR). Reactions were run using 1 μL cDNA in PowerSYBR Green PCR Master Mix (ThermoFisher), and 0.2 pM of each primer. Reactions were performed in a StepOne Real-Time System (ThermoFisher) under conditions of initial denaturation at 95°C for 10 mins followed by 40 cycles of 95°C for 15 secs, and 60°C for 1 min. CT values from the triplicate runs were averaged and analyzed by using the 2^−ΔΔCt^ method.

### Genomic DNA extraction

For cells grown in tissue culture, the cells were initially harvested by treating in 0.25% Trypsin-EDTA (Gibco), with washing twice in 1xPBS and final centrifugation at 1,000 rpm for four mins. Cells were diluted to a final concentration of 1x10^5^ using a Countess II (ThermoFisher) and subjected to genomic DNA extraction using a Nucleospin DNA extraction kit (Machery-Nagel) according to the manufacturer’s protocol. Briefly, samples were lysed using Proteinase K, washed in ethanol, and eluted in DNAse-free water. Genomic DNA was extracted from tumor and cryopreserved cells as above with the modification that tumor tissues were cut in 25 mg sections and further cut into small pieces before cellular lysis, while 500 μl of tissue was used. Genomic DNA was successfully extracted from a total of 29 of the 33 samples and the products used in PCR-based genotyping.

### Genotyping

Genotyping was performed using PCR screens of Fc1(a) insertions previously characterized in modern breeds [21] at a total of 54 sites (*i*.*e*., requirements of 5’ and 3’ LTR with clear breakpoints and presence of matched target site duplications) (S1 Table). These loci included 14 Fc1(a) previously confirmed to possess a full-length provirus allele. Insertions were excluded under the criteria of ones missing either of the two LTR-genome junctions or that had evidence of location within an encompassing segmental duplication [21]. To detect the presence of a full-length provirus, an internal primer near base ~506 (within the internal 5’ untranslated region; 5’ GAGAGAGCCTCCGTGCTGTTG) from the start of the Fc1(a)_CON_ element was paired with a locus-specific flanking primer, accounting for insertion orientation. A second PCR was run with primer pairs flanking each insertion locus to detect the presence of a solo-LTR or unoccupied site. Reactions were performed using 0.125 U *Taq* polymerase (Invitrogen) in 10xbuffer, 2.5 μM dNTPs, 10 μM either primer, and 2.5 μM MgCl_2_ under the conditions of initial denaturation at 95°C for 2 mins followed by 35 cycles of 95°C for 30 secs, 59°C for 30 secs, 72°C for 1:15 mins, and a final extension at 72°C for 3 mins. A volume of 10 μL of the PCR reaction was visually assessed by electrophoreses in 1% agarose in 1xTBE. Genotypes were manually scored and accounted for insertions allelic presence of either provirus and/or solo-LTR. Raw genotype data is provided in S1 Data.

## Supporting information

S1 Fig LTR-based phylogeny of Fc1(a) loci in the study. Neighbor-joining tree of Fc1(a) LTRs for insertions analyzed in this study. Nucleotide sequences was aligned using MUSCLE [39] and edited using BioEdit [35]. A phylogeny was then reconstructed by the neighbor-joining method in MEGA [40] using the Kimura 2-parameter model and gamma distribution of 2.5 and 100 replicates. Proviruses are denoted by presence of 5’ and/or 3’ LTR. The black envelope symbols show placement of proviruses with *env* ORFs, with shading used to indicate un-paired LTRs of the same provirus; a gray envelope indicates the *env* reading frame is interrupted; gray boxed triangles indicate proviruses having deletion including the *env* gene.
(TIF)

S2 Fig Properties of the Fc1(a) consensus provirus and region targeted in the study. (A) A schematic representation of an *in silico* consensus provirus deduced from 19 full-length elements (11 and 8 proviruses present or absent in CanFam3.1, respectively). The *gag* product contains predicted functional regions for the matrix (MA), capsid (CA), and nucleocapsid (NC) domains; the *pro*/*pol* product contains conserved motifs for protease (PRO), reverse transcriptase (RT), RNase H, and integrase (IN); the *env* product possesses a furin cleavage site (RRKR), as well as predicted RD114-and-D-type (RDR) receptor binding motif, as well as CWIC (SU) and CX_6_CC (TM) motifs involved in SU-TM interactions [21]. Consistent with other γ-like retroviruses, the *env* ORF resides within an alternate reading frame overlapping the 3’ end of the *pro*/*pol* gene. Annotated deletions including *env* are indicated in gray; the region targeted in the study is indicated in black [23]. (B) Primer sequences and target region used in the study. Aligned sequences of the target region are shown for *env*-containing Fc1(a) proviruses. ‘Ref’ indicates presence in CamFam3.1; ‘nonRef’ loci are empty in CanFam3.1. The start positions of annotated deletions are indicated by gray arrows.
(TIF)

S1 Table Summarization of Fc1(a) loci and properties in the study. Information for Fc1(a) loci used in this study including ORF presence and gene intersections. The coordinate positions given are relative to CanFam3.1.
(XLSX)

S2 Table Demographic information for dogs included in the study. Information includes breed, sex, neuter status, and age at diagnosis or when the sample was obtained, as available; any mix breed backgrounds are indicated as “MIX”.
(XLSX)

S1 Data Raw genotype data for tumor samples and cell lines. Raw genotype data obtained across tissues from 29 dogs and 4 cell lines. Genotypes are written as 0 (-/-), 0.5 (-/+), or 1 (+/+). An S or P indicates solo-LTR or provirus. All other provirus sites for which an insertion was detected reflect the presence of the full-length allele.
(XLSX)

## References

[1] Jern P, Coffin JM. Effects of retroviruses on host genome function. Annual review of genetics. 2008;42:709–32. doi: 10.1146/annurev.genet.42.110807.091501 18694346

[2] Sverdlov ED. Retroviruses and primate genome evolution. Georgetown, Tex.: Landes Bioscience; 2005. 250 p. p.

[3] Coffin JM, Hughes SH, Varmus H. Retroviruses. Plainview, N.Y.: Cold Spring Harbor Laboratory Press; 1997. xv, 843 p. p. 21433340

[4] Swanstrom R, Wills JW. Synthesis, Assembly, and Processing of Viral Proteins. In: Coffin JM, Hughes SH, Varmus HE, editors. Retroviruses. Cold Spring Harbor (NY) 1997. 21433349

[5] Wildschutte JH, Williams ZH, Montesion M, Subramanian RP, Kidd JM, Coffin JM. Discovery of unfixed endogenous retrovirus insertions in diverse human populations. Proceedings of the National Academy of Sciences of the United States of America. 2016;113(16):E2326–34. doi: 10.1073/pnas.1602336113 27001843 PMC4843416

[6] Johnson WE. Origins and evolutionary consequences of ancient endogenous retroviruses. Nat Rev Microbiol. 2019;17(6):355–70. doi: 10.1038/s41579-019-0189-2 30962577

[7] Chapman AL, Bopp WJ, Brightwell AS, Cohen H, Nielsen AH, Gravelle CR, et al. Preliminary report on virus-like particles in canine leukemia and derived cell cultures. Cancer Res. 1967;27(1):18–25. 6020359

[8] Ghernati I, Auger C, Chabanne L, Corbin A, Bonnefont C, Magnol JP, et al. Characterization of a canine long-term T cell line (DLC 01) established from a dog with Sezary syndrome and producing retroviral particles. Leukemia. 1999;13(8):1281–90. 10450758 10.1038/sj.leu.2401480

[9] Ghernati I, Corbin A, Chabanne L, Auger C, Magnol JP, Fournel C, et al. Canine large granular lymphocyte leukemia and its derived cell line produce infectious retroviral particles. Veterinary pathology. 2000;37(4):310–7. doi: 10.1354/vp.37-4-310 10896392

[10] Perk K, Safran N, Dahlberg JE. Propagation and characterization of novel canine lentivirus isolated from a dog. Leukemia. 1992;6 Suppl 3:155S–7S. 1376381

[11] Safran N, Perk K, Eyal O, Dahlberg JE. Isolation and preliminary characterisation of a novel retrovirus isolated from a leukaemic dog. Res Vet Sci. 1992;52(2):250–5. doi: 10.1016/0034-5288(92)90018-w 1374929

[12] Onions D. RNA-dependent DNA polymerase activity in canine lymphosarcoma. Eur J Cancer. 1980;16(3):345–50. doi: 10.1016/0014-2964(80)90351-5 6154578

[13] Tomley FM, Armstrong SJ, Mahy BW, Owen LN. Reverse transcriptase activity and particles of retroviral density in cultured canine lymphosarcoma supernatants. Br J Cancer. 1983;47(2):277–84. doi: 10.1038/bjc.1983.36 6186265 PMC2011285

[14] Modiano JF, Getzy DM, Akol KG, Van Winkle TJ, Cockerell GL. Retrovirus-like activity in an immunosuppressed dog: pathological and immunological findings. Journal of comparative pathology. 1995;112(2):165–83. doi: 10.1016/s0021-9975(05)80059-3 7539463

[15] Sykes GP, King JM, Cooper BC. Retrovirus-like particles associated with myeloproliferative disease in the dog. Journal of comparative pathology. 1985;95(4):559–64. doi: 10.1016/0021-9975(85)90025-8 4067023

[16] Lombard LS, Moloney JB, Rickard CG. Transmissible Canine Mastocytoma. Ann N Y Acad Sci. 1963;108:1086–105. doi: 10.1111/j.1749-6632.1963.tb13437.x 14081493

[17] Stocking C, Kozak CA. Murine endogenous retroviruses. Cellular and molecular life sciences: CMLS. 2008;65(21):3383–98. 18818872 10.1007/s00018-008-8497-0 PMC4802364

[18] Anai Y, Ochi H, Watanabe S, Nakagawa S, Kawamura M, Gojobori T, et al. Infectious endogenous retroviruses in cats and emergence of recombinant viruses. J Virol. 2012;86(16):8634–44. doi: 10.1128/JVI.00280-12 22674983 PMC3421742

[19] Lindblad-Toh K, Wade CM, Mikkelsen TS, Karlsson EK, Jaffe DB, Kamal M, et al. Genome sequence, comparative analysis and haplotype structure of the domestic dog. Nature. 2005;438(7069):803–19. doi: 10.1038/nature04338 16341006

[20] Barrio AM, Ekerljung M, Jern P, Benachenhou F, Sperber GO, Bongcam-Rudloff E, et al. The first sequenced carnivore genome shows complex host-endogenous retrovirus relationships. PloS one. 2011;6(5):e19832. doi: 10.1371/journal.pone.0019832 21589882 PMC3093408

[21] Halo JV, Pendleton AL, Jarosz AS, Gifford RJ, Day ML, Kidd JM. Origin and recent expansion of an endogenous gammaretroviral lineage in domestic and wild canids. Retrovirology. 2019;16(1):6. doi: 10.1186/s12977-019-0468-z 30845962 PMC6407205

[22] Diehl WE, Patel N, Halm K, Johnson WE. Tracking interspecies transmission and long-term evolution of an ancient retrovirus using the genomes of modern mammals. Elife. 2016;5:e12704. doi: 10.7554/eLife.12704 26952212 PMC4798954

[23] Sinha A, Johnson WE. Retroviruses of the RDR superinfection interference group: ancient origins and broad host distribution of a promiscuous Env gene. Curr Opin Virol. 2017;25:105–12. doi: 10.1016/j.coviro.2017.07.020 28837888

[24] Boeke JD, Stoye JP. Retrotransposons, endogenous retroviruses, and the evolution of retroelements. In: Coffin J, Hughes S, Varmus H, editors. Retroviruses. New York, NY: CSHL Press; 1997. p. 343–435. 21433351

[25] Halo JV, Pendleton AL, Shen F, Doucet AJ, Derrien T, Hitte C, et al. Long-read assembly of a Great Dane genome highlights the contribution of GC-rich sequence and mobile elements to canine genomes. Proceedings of the National Academy of Sciences of the United States of America. 2021;118(11). doi: 10.1073/pnas.2016274118 33836575 PMC7980453

[26] Okubo M, Tsurukubo Y, Higaki T, Kawabe T, Goto M, Murase T, et al. Clonal chromosomal aberrations accompanied by strong telomerase activity in immortalization of human B-lymphoblastoid cell lines transformed by Epstein-Barr virus. Cancer Genet Cytogenet. 2001;129(1):30–4. doi: 10.1016/s0165-4608(01)00420-4 11520562

[27] Cohen CJ, Lock WM, Mager DL. Endogenous retroviral LTRs as promoters for human genes: a critical assessment. Gene. 2009;448(2):105–14. doi: 10.1016/j.gene.2009.06.020 19577618

[28] Salmons B, Gunzburg WH. Current perspectives in the biology of mouse mammary tumour virus. Virus Res. 1987;8(2):81–102. doi: 10.1016/0168-1702(87)90022-0 2823501

[29] Cornelis G, Vernochet C, Malicorne S, Souquere S, Tzika AC, Goodman SM, et al. Retroviral envelope syncytin capture in an ancestrally diverged mammalian clade for placentation in the primitive Afrotherian tenrecs. Proceedings of the National Academy of Sciences of the United States of America. 2014;111(41):E4332–41. doi: 10.1073/pnas.1412268111 25267646 PMC4205661

[30] Kitao K, Sumiyochi A, Nakagawa S, Matsumoto Y, Mizuno T, Miyazawa T. Systematic Identification of Endogenous Retroviral Protein-Coding Genes Expressed in Canine Oral Malignant Melanoma. Frontieers in Virology. 2021;24.

[31] Mager DL, Stoye JP. Mammalian Endogenous Retroviruses. Microbiol Spectr. 2015;3(1):MDNA3-0009–2014. doi: 10.1128/microbiolspec.MDNA3-0009-2014 26104559

[32] Flockerzi A, Ruggieri A, Frank O, Sauter M, Maldener E, Kopper B, et al. Expression patterns of transcribed human endogenous retrovirus HERV-K(HML-2) loci in human tissues and the need for a HERV Transcriptome Project. BMC Genomics. 2008;9:354. doi: 10.1186/1471-2164-9-354 18664271 PMC2525661

[33] Frank O, Giehl M, Zheng C, Hehlmann R, Leib-Mosch C, Seifarth W. Human endogenous retrovirus expression profiles in samples from brains of patients with schizophrenia and bipolar disorders. J Virol. 2005;79(17):10890–901. doi: 10.1128/JVI.79.17.10890-10901.2005 16103141 PMC1193590

[34] Ochi Y. Genetic landscape of chronic myeloid leukemia. Int J Hematol. 2023;117(1):30–6. doi: 10.1007/s12185-022-03510-w 36477676

[35] Hall TA. BioEdit: a user-friendly biological sequence alignment editor and analysis program for Windows 95/98/NT. Nucleic acids symposium series. 1999;41:95–8.

[36] Fenger JM, Roberts RD, Iwenofu OH, Bear MD, Zhang X, Couto JI, et al. MiR-9 is overexpressed in spontaneous canine osteosarcoma and promotes a metastatic phenotype including invasion and migration in osteoblasts and osteosarcoma cell lines. BMC Cancer. 2016;16(1):784. doi: 10.1186/s12885-016-2837-5 27724924 PMC5057229

[37] Hao Z, Lv D, Ge Y, Shi J, Weijers D, Yu G, et al. RIdeogram: drawing SVG graphics to visualize and map genome-wide data on the idiograms. PeerJ Comput Sci. 2020;6:e251. doi: 10.7717/peerj-cs.251 33816903 PMC7924719

[38] Young AC, Kirkness EF, Breen M. Tackling the characterization of canine chromosomal breakpoints with an integrated in-situ/in-silico approach: the canine PAR and PAB. Chromosome Res. 2008;16(8):1193–202. doi: 10.1007/s10577-008-1268-9 19005636

[39] Edgar RC. MUSCLE: multiple sequence alignment with high accuracy and high throughput. Nucleic Acids Res. 2004;32(5):1792–7. doi: 10.1093/nar/gkh340 15034147 PMC390337

[40] Kumar S, Stecher G, Tamura K. MEGA7: Molecular Evolutionary Genetics Analysis Version 7.0 for Bigger Datasets. Mol Biol Evol. 2016;33(7):1870–4. doi: 10.1093/molbev/msw054 27004904 PMC8210823

