# Supplementary figures and images for: Expression and high levels of insertional polymorphism of an endogenous gammaretrovirus lineage in dogs
Source: PLoS Genet. 2023 Dec 6;19(12):e1011083. doi: 10.1371/journal.pgen.1011083 (PMC10727363; doi:10.1371/journal.pgen.1011083)

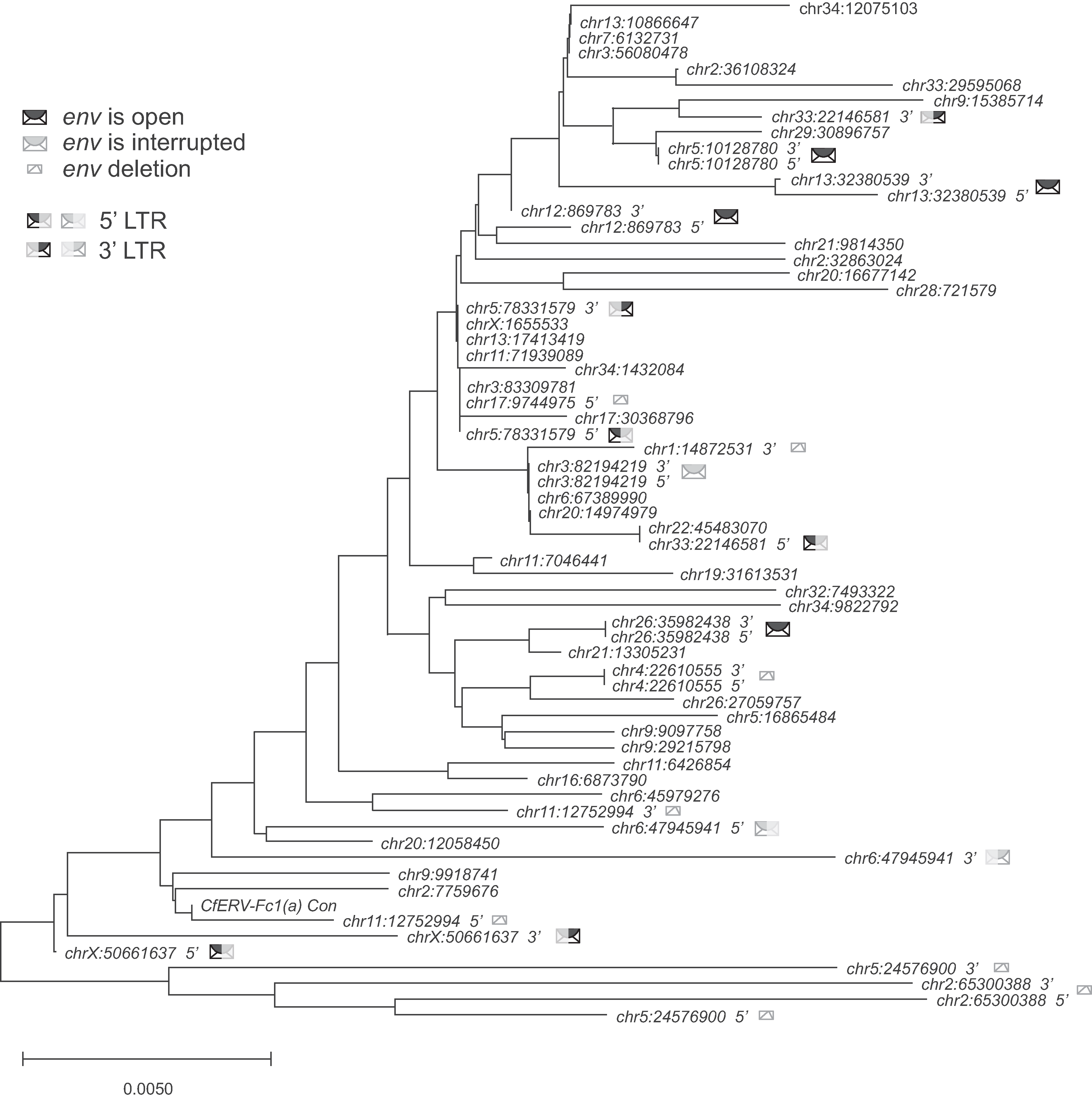

Supplement: S1 Fig — Neighbor-joining tree of Fc1(a) LTRs for insertions analyzed in this study. Nucleotide sequences was aligned using MUSCLE [39] and edited using BioEdit [35]. A phylogeny was then reconstructed by the neighbor-joining method in MEGA [40] using the Kimura 2-parameter model and gamma distribution of 2.5 and 100 replicates. Proviruses are denoted by presence of 5’ and/or 3’ LTR. The black envelope symbols show placement of proviruses with env ORFs, with shading used to indicate un-paired LTRs of the same provirus; a gray envelope indicates the env reading frame is interrupted; gray boxed triangles indicate proviruses having deletion including the env gene. (TIF) [file pgen.1011083.s001.tif]

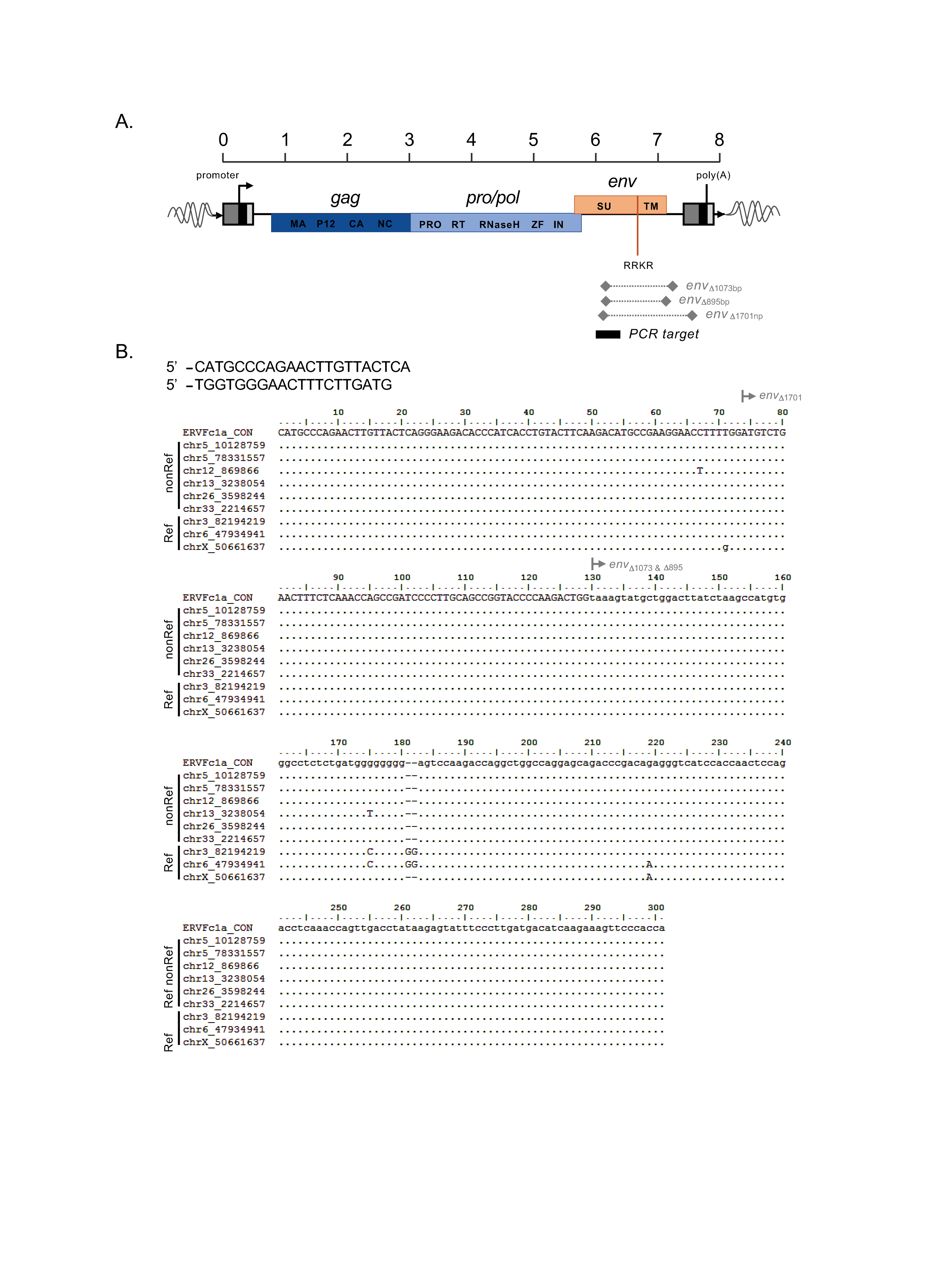

Supplement: S2 Fig — (A) A schematic representation of an in silico consensus provirus deduced from 19 full-length elements (11 and 8 proviruses present or absent in CanFam3.1, respectively). The gag product contains predicted functional regions for the matrix (MA), capsid (CA), and nucleocapsid (NC) domains; the pro/pol product contains conserved motifs for protease (PRO), reverse transcriptase (RT), RNase H, and integrase (IN); the env product possesses a furin cleavage site (RRKR), as well as predicted RD114-and-D-type (RDR) receptor binding motif, as well as CWIC (SU) and CX6CC (TM) motifs involved in SU-TM interactions [21]. Consistent with other γ-like retroviruses, the env ORF resides within an alternate reading frame overlapping the 3’ end of the pro/pol gene. Annotated deletions including env are indicated in gray; the region targeted in the study is indicated in black [23]. (B) Primer sequences and target region used in the study. Aligned sequences of the target region are shown for env-containing Fc1(a) proviruses. ‘Ref’ indicates presence in CamFam3.1; ‘nonRef’ loci are empty in CanFam3.1. The start positions of annotated deletions are indicated by gray arrows. (TIF) [file pgen.1011083.s002.tif]
